# Cross-sectional ocean current data from vessel mounted acoustic Doppler current profiler (ADCP) survey in the Alas Strait, Indonesia

**DOI:** 10.1016/j.dib.2025.111576

**Published:** 2025-04-21

**Authors:** Sendy Brammadi, Poerbandono, Roberto Mayerle

**Affiliations:** a Master Program in Geodesy and Geomatics Engineering, Faculty of Earth Sciences and Technology, Institut Teknologi Bandung, Jalan Ganesha 10, 40132 Bandung, Indonesia; b Hydrography Research Group, Faculty of Earth Sciences and Technology, Institut Teknologi Bandung, Jalan Ganesha 10, 40132 Bandung, Indonesia; c Research and Technology Centre Westcoast (FTZ), University of Kiel, Otto-Hahn-Platz 3, 24118 Kiel, Germany

**Keywords:** Three-dimensional current profile, Spring tide, Tidal elevation, Bathymetry

## Abstract

In this article, we present three-dimensional current vectors obtained by 600 kHz vessel mounted Teledyne Workhorse Acoustic Doppler Current Profiler (ADCP). The data is collected during a field measuring campaign on the 25th to the 27th of September 2022 (spring tide) in the Alas Strait, Indonesia. The data presented is the first in-situ measurement in this region to collect ocean current data from a vessel moving across multiple cross-sectional transects. The collected ADCP raw data undergoes conversion to ASCII format, reformatting into a more organized tabulation, and quality control for removal of bad data due to biases, errors, interferences, and/or noises. Moreover, tidal and depth data are also provided. As many as 13 cross-sectional transects and one along strait transect are presented covering water depth from about 1.71 m to approximately 60 m. These current, tide, and depth datasets provide an opportunity to characterize current behaviour at specific tidal phases and locations. The cross-sectional observation could be used to calibrate and validate results from hydrodynamic models.

Specifications Table

**Table:** 

Subject	Physical oceanography
Specific subject area	Ocean current observation
Data format	Measurement file (.mmt), ADCP observation data (.PD0), Converted ADCP data (.TXT), filtered ADCP data and tide data (.TXT, .CSV), and geo-image (.TIFF)
Type of data	1. Measurement files (.mmt): Files containing field configuration of each cross-sectional transect. 2. ADCP observation data (.PD0): Files containing all observables recorded by the ADCP, including their positions. 3. ASCII files (.TXT, .CSV): Files containing the reformatted data resulted from the conversion of and filtered ADCP data stored as .TXT and tide data in .CSV file format. 4. Geo-image: Georeferenced digital elevation model (DEM) image.
Data collection	Data are collected using a Teledyne Workhorse Monitor 600 kHz ADCP controlled by WinRiver II software. The ADCP is deployed downward mounted from the Motor Vessel (MV) Tenggara Supporter. The position is obtained from the Real-Time Precise Point Positioning (RT-PPP) GNSS device described as latitude and longitude in World Geodetic System 1984 (WGS84). The collected data is organized into 14 files representing 13 cross-sectional transects (with an average length of 10 km and 1 km spacing between transects) and one along strait transect. The vessel navigation is guided with the help of by Hypack 2014 software. Tidal elevation during the survey is generated from TPXO-9 model from the centre of the survey location (i.e., 8° 37′ 30.36" S and 116° 41′ 57.48" E). The depths are generated from the national bathymetric model dataset provided by the Indonesian Geospatial Agency.
Data source location	Alas Strait, between Lombok and Sumbawa islands, Indonesia.Approximate survey site boundaries (WGS 1984 datum): 1. North: 008° 34′ 15" S 2. South: 008° 41′ 27" S 3. West: 116° 37′ 41" E 4. East: 116° 45′ 34" E
Data accessibility	The dataset is available at Mendeley data repository with details as follow. Data identification number: 10.17632/cwcbfjvwzz.3 Direct URL to data: https://data.mendeley.com/datasets/cwcbfjvwzz/3 Instructions for accessing these data: The direct URL provided above facilitate straight access to the data. After finding the file available, simply click “Download” to access the data, which is compressed in a format .zip

## 1 Value of the Data

• Among the first in-situ measurement in Alas Strait collected from a moving vessel across multiple cross-sectional transects.
• Can be used to characterize currents’ spatial variation across the channel and temporal evolution in different tidal phase.
• Can be used for the calibration and validation of results from hydrodynamic models in three-dimensional model.

## 2 Background

The Alas strait is of importance due to its role as one of the minor outlets of the Indonesian throughflow and the potential of tidal power generation [1,2]. Ocean current data presented here is acquired from an *in-situ* field survey and supplemented with additional information (from secondary sources) comprising of the tide (over the period of the survey) and bathymetry representing the survey area. The motivation of the *in-situ* ocean current acquisition is to provide spatial coverage of current patterns enabling further analysis for describing the characteristics and behaviour on how they vary with space and time. The *in-situ* field survey is carried out with a down-ward looking vessel-mounted acoustic Doppler current profiler (VM-ADCP). The survey is done in 13 cross-sectional transects perpendicular to the strait and one cross-sectional transect parallel to the strait. Additional information consists of the bathymetry of the survey area and record of tidal observation acquired from the public repositories.

## 3 Data Description

**Fig. 1:**
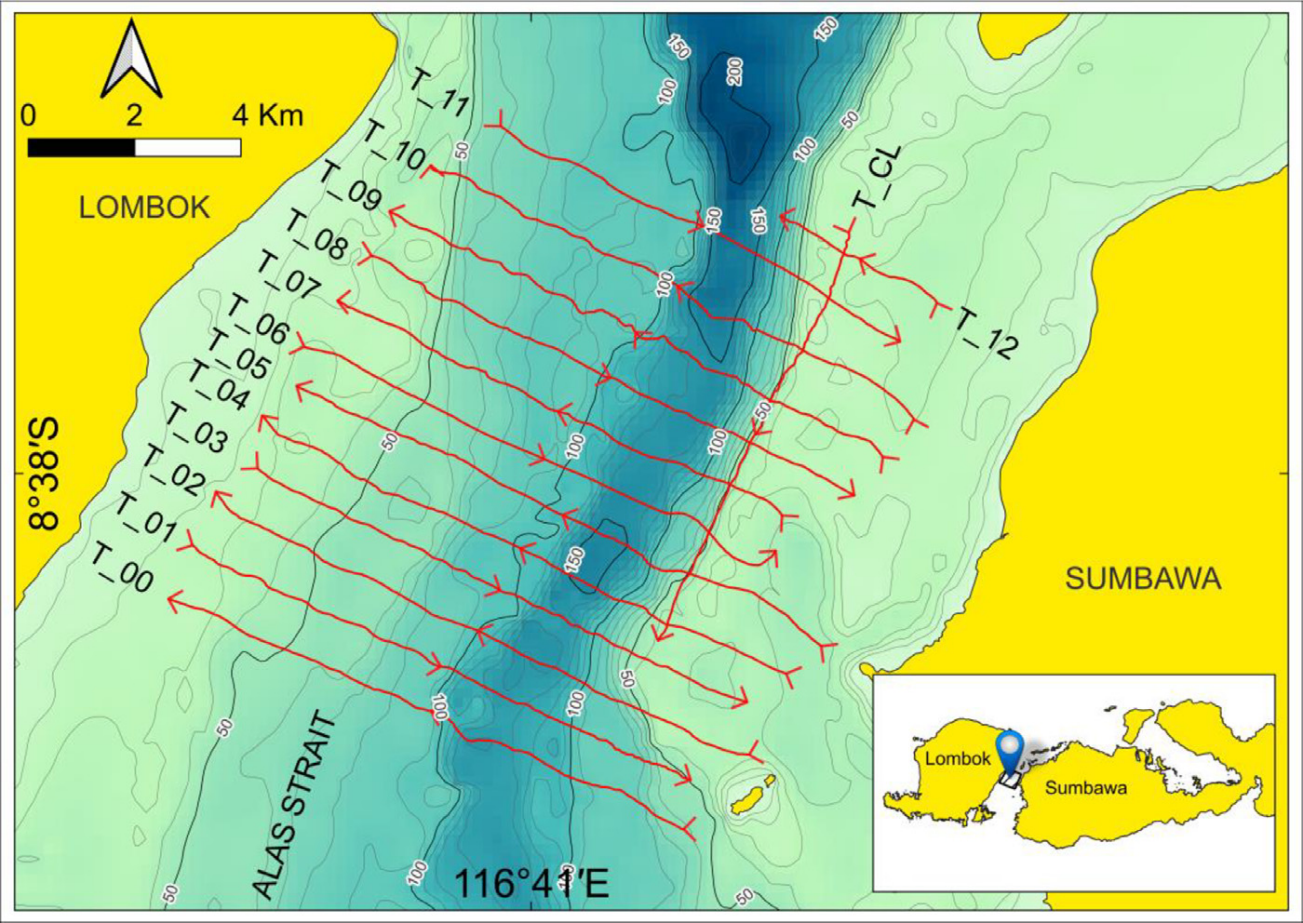
Vessel route on the transect line at the survey site. The label in left of lines indicates the order of the transect name (abbreviated as T). Arrowheads indicate survey direction.

The VM ADCP data is collected the 25th to the 27th of September 2022. It consists of 14 files corresponding to a cross-sectional transect. The typical length of the transects 10 km and with 1 km spacing. An exception is the transect 12 as it covers only about 3 km. The set of data submitted here consists of ADCP data (i.e., raw and pre-processed), tidal data, and bathymetry. Table 1 provides an overview of the dataset organized in four different folders. Folder “1_Native_ADCP_Data” contains the results of ADCP measurements in their native format. The measurements configuration used during data acquisition are stored per day in the .mmt file extension and used for ADCP data collection on that day. The ADCP observation results of each transect are stored with the .PD0 extension at same folder. The measurement files used per day, the ADCP observation results of each transect on that particular day and the corresponding transect names are shown in Table 2. Folders “2_Converted_ADCP_Data” and “3_Filtered_ADCP_and_Tide_Data” contain data in ASCII format as they are described in Table 1. The converted ADCP data is reformatted as an effort of distinguish different observables into 16 different columns (Table 3). Folder 4_Bathymetry_Data” contains gridded bathymetric data. Visual presentation of the bathymetry and the observed cross-sectional transects is given in Fig. 1. Coastline, geographic names, and cartographic design shown in Fig. 1 are not included in this submission.

**Table 1:** Content of the related field dataset (DOI: 10.17632/cwcbfjvwzz.3).

Folder Name	Content of Folder
1_ Native ADCP_Data	a. Three measurement files (.mmt) contains configurations used to collect ADCP data, such as ADCP transducer draught, data connection and data storage. The naming of the measurement files is based on the day of measurement. b. Fifteen ADCP observation file (.PD0) contains all data sent from ADCP during data collection. The naming of the observation file is based on day of measurement and file sequence of record data. These files comprising of 13 files from cross-sectional transects (one file .PD0 stores one cross-sectional transect), one cross line transect (along the strait), and one test. c. Fifteen navigation file (.TXT) contains position data received during the ADPC data acquisition. The naming of navigation file follows the name of ADCP observation file. These files are not used in data processing. Details of the daily measurement files used for ADCP data collection and the corresponding transect names are shown in Table 2.
2_Converted_ADCP_Data	ASCII or .TXT files from all transects. There are 14 files inside this folder. These files are generated directly by converting the native ADCP data into ASCII format with the help of with ADCP’s Winriver II software. During the conversion, the file names were renamed according to the name of the cross-sectional transects (Table 2). The ASCII files resulted from the conversion contain velocity profile in each observation time or epoch termed as ensemble.
3_Filtered_ADCP_and_Tide_Data	Fourteen filtered ADCP transect data in ASCII format stored as .TXT files and one tidal data also in ASCII format stored as Tide_Alas.CSV. Details of the ADCP and tide data in this folder are described herein.
	(a) In the filtered ADCP transect data, the so-called “bad data” are removed. Bad data is identified by the ADCP software algorithm due to biases, errors, interferences, and/or noises. Details of the tabulated format of such data are described in Table 3.
	(b) Tide data is stored in tabular form as .CSV file format and the tide visualization can be seen in Fig. 2.
4_Bathymetry_Data	Gridded depth data around the survey area expressed as meter with respect to the mean sea level, with the geodetic position described as latitude and longitude in WGS84 datum.

**Table 2:** Daily measurement files used during ADCP data collection, file observation, and corresponding transect names.

Measurement File	ADCP Observation File	Transect Name
RG_2022.09-25.0.mmt	RG_2022-09-25_0_000.PD0	Equipment Test
	RG_2022-09-25_0_001.PD0	Transect 05
	RG_2022-09-25_0_002.PD0	Transect 06
	RG_2022-09-25_0_003.PD0	Transect 07
	RG_2022-09-25_0_004.PD0	Transect 08
	RG_2022-09-25_0_005.PD0	Transect 09
RG_2022.09-26.0.mmt	RG_2022-09-26_0_000.PD0	Transect 10
	RG_2022-09-26_0_001.PD0	Transect 11
	RG_2022-09-26_0_002.PD0	Transect 12
	RG_2022-09-26_0_003.PD0	Transect Cross Line
RG_2022.09-27.1.mmt	RG_2022-09-27_1_000.PD0	Transect 04
	RG_2022-09-27_1_001.PD0	Transect 03
	RG_2022-09-27_1_002.PD0	Transect 02
	RG_2022-09-27_1_003.PD0	Transect 01
	RG_2022-09-27_1_004.PD0	Transect 00

**Table 3:** ASCII header description of the data stored in the folder 3_Filtered_ADCP_and_Tide_Data.

Column	Header	Parameter	Unit
***Filtered ADCP file (.TXT)***			
1	date	Ensemble Time	UTC^a^
2	ens	Ensemble Number	-
3	dist	Total Elapsed Distance Through This Ensemble	meter
4	lat	Latitude of Ensemble	decimal degree
5	lon	Longitude of Ensemble	decimal degree
6	roll	ADCP Roll Moving Attitude of Ensemble	degree
7	pitch	ADCP Pitch Moving Attitude of Ensemble	degree
8	h	Depth From average 4 beam reading	meter
9	hb	Depth For Present Bin	meter
10	u	East West Vector Velocity Magnitude	m/s
11	v	North South Vector Velocity Magnitude	m/s
12	w	Up Down Vector Velocity Magnitude	m/s
13	errv	Variance Error Velocity	m/s
14	vel	Total Velocity Magnitude	m/s
15	dir	Total Velocity Direction	decimal degree
16	bs	Backscatter From Average 4 Beam reading	decibel (db)
***Tide data (Tide Alas.CSV)***			
1	Time	Datapoint Time	UTC^1^
2	Height	Height of tidal elevation above mean sea level	meter

a Universal Time Coordinated. Time at longitude 0° or the prime meridian.

**Fig. 2:**
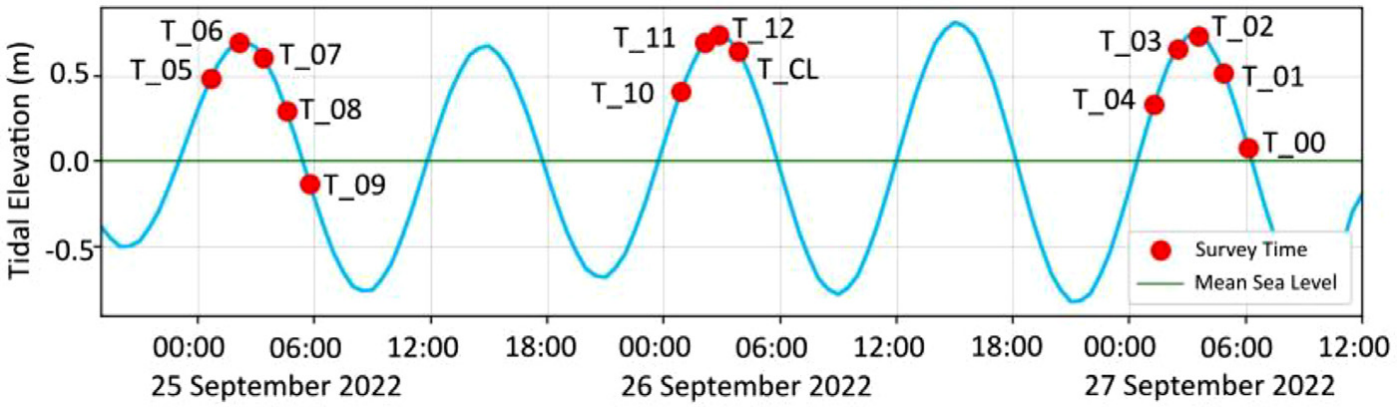
Overview of tide level data and overlay with centre time of transects line. The label next to the red dot indicates the transect name (abbreviated as T) at the corresponding time.

## 4 Experimental Design, Materials and Methods

**Fig. 3:**
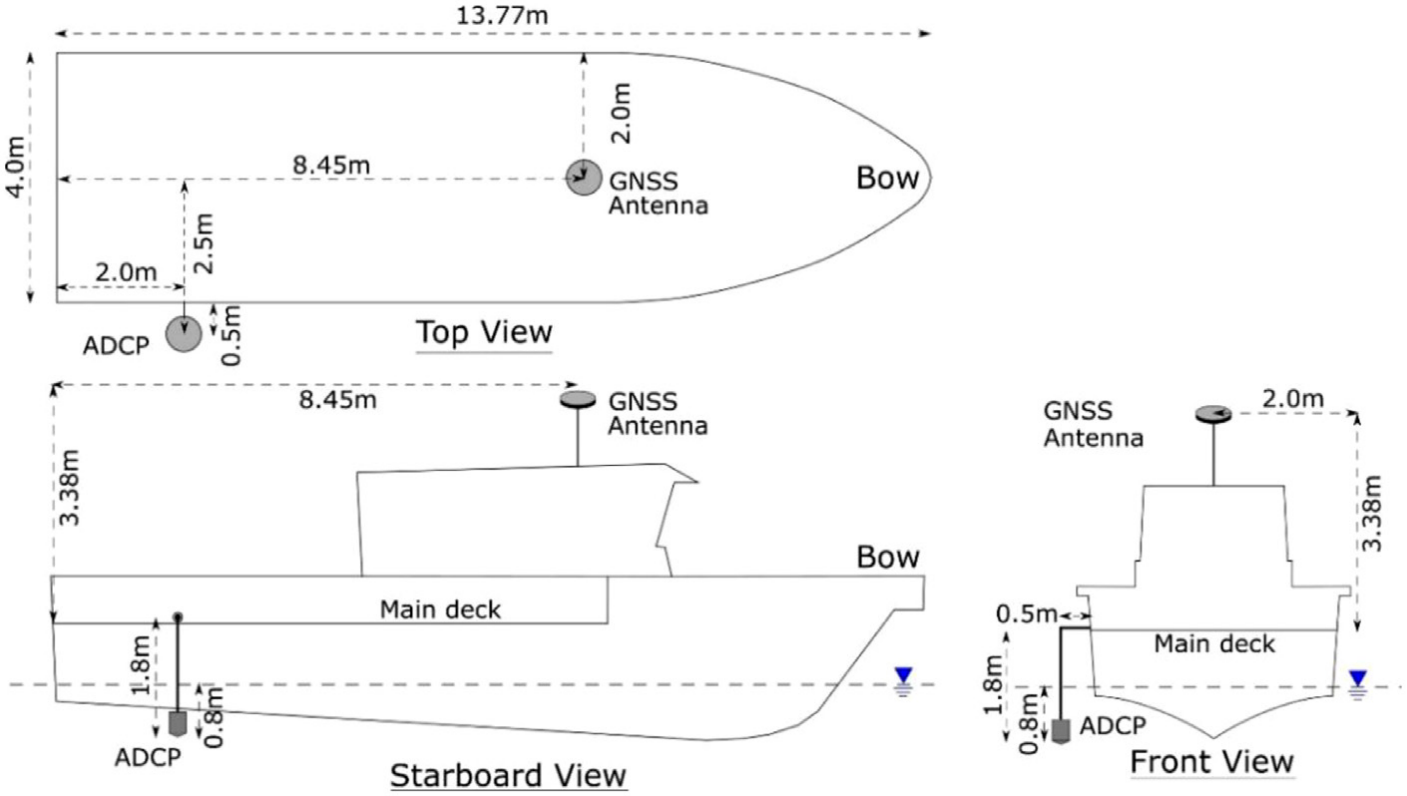
Vessel offset diagram of equipment used onboard MV Tenggara Supporter.

1. Installation and instrument configuration
  A 600 kHz Teledyne Workhorse ADCP with bottom tracking capability and 2 Hz of sampling rate is deployed from Motor Vessel (MV) Tenggara Supporter, controlled on-board with WinRiver II software. The ADCP is mounted with a pole at the starboard side of MV Tenggara Supporter with draught of 0.8 m from the water surface. The blanking distance from the face of ADCP sensor is 0.91 cm with vertical resolution of the observed currents cell is 0.5 m, the configuration set on the ADCP is capable of covering the water column from about 1.71 m below the surface of water down up to approximately 60 m depth. The vessel is navigated with the aid of a Trimble R9 Global Navigation Satellite System (GNSS) in Real-Time Precise Point Positioning mode controlled by Hypack 2014 software. The approximate positioning accuracy is within the cm-level [3]. The geographic coordinates refer to World Geodetic System (WGS) 1984. The GNSS antenna is affixed on the top of the bridge’s deck. The lateral offsets of the ADCP’s pole with respect to the position of the GNSS antenna is measured and injected into the WinRiver II software as constants. Fig. 3 shows the dimensional information of the installation diagram of the ADCP, including the lateral offsets of the ADCP’s pole with respect to the position of the GNSS antenna. In Fig. 4 impressions on the mounting of the pole and the monitoring ADCP data by the technicians and on-board operators are given.

**Fig. 4:**
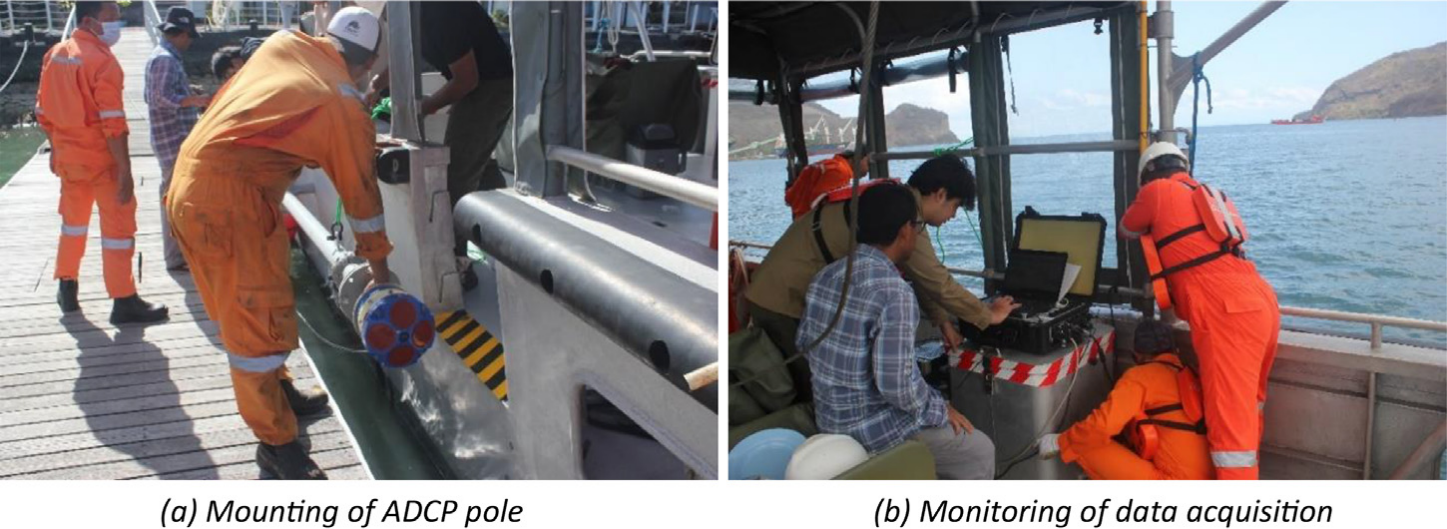
Installation and operation of ADCP.
2. Sequence of data acquisition
  Prior to the acquisition, the ADCP is configured with the help of WinRiver II software. The configuration includes primarily the vertical and horizontal offsets respectively due to the installation of the ADCP transducer and the positioning device, i.e., GNSS. In addition to that, the pre-survey quality assurance is applied. This comprises of the synchronization of time, offsetting the pressure gauge (for the monitoring of vessel’s heave and settlement), and heading alignment of ADCP compass. The configuration of the acquisition system is stored in the daily measurement file as .mmt format. The raw ADCP data acquired during the field observation are converted into ASCII from their native format (i.e., .PD0) and stored as .TXT files. From here on, the observed currents along the cross sectional transect are organized into multiple ensembles corresponding to a measuring column identified by unique time and position tags. Following this, additional sequence is applied to remove the so-called “bad data”. Bad data is a signature identified by a specific algorithm in the ADCP software algorithm to tag unreliable observations due to biases, errors, interferences, and/or noises [4]. The unreliable data is flagged by ADCP software with specific values such as -32,768 for velocity, 2147483647 for discharge, and 30,000 for position [5]. The final data format is detailed in Table 2.
3. Peripheral information
  Hourly basis elevation of tide throughout the period of the survey is generated from the TPXO-9^1^ and acquired with the help of Tide Model Driver (TMD) toolbox^2^ on the Matlab software [6] which publicly available. The point of interest is located at the centre of the survey site, i.e., 8° 37′ 30.36" S and 116° 41′ 57.48" E. One-minute tidal elevation data is interpolated from the hourly data to allow tide-current time synchronization. The water depths are obtained from the national bathymetry model dataset^3^ and has a spatial resolution of 6 arc-s (∼185 m at the equator) [7]. The gridded depth data expressed as meter and referenced to the mean sea level. The horizontal position is described as latitude and longitude of the World Geodetic System 1984 (WGS).

## Limitations

The high-frequency ADCP device used in the survey is capable of recording high resolution data but limited in its penetration range. The coverage is hence limited to about 60 m depth. Due to safety reasons and logistical constraints, the data acquisition can only be carried out during the daylight.

## Ethics Statement

The authors have read and followed the ethical requirements for publication in Data in Brief, and this work does not involve human subjects, animal experiments, or any data collected from social media platforms.

## CRediT authorship contribution statement

**Sendy Brammadi:** Data curation, Visualization, Writing – original draft. **Poerbandono:** Supervision, Writing – review & editing. **Roberto Mayerle:** Supervision, Writing – review & editing.

## Data Availability

Mendeley DataAlas Strait Vessel Mounted ADCP Dataset (Original data)

## Footnotes

1 https://www.tpxo.net/global.

2 https://www.github.com/EarthAndSpaceResearch/TMD_Matlab_Toolbox_v2.5.

3 https://tanahair.indonesia.go.id/demnas/.
